# Combination treatment with Grb7 peptide and Doxorubicin or Trastuzumab (Herceptin) results in cooperative cell growth inhibition in breast cancer cells

**DOI:** 10.1038/sj.bjc.6603732

**Published:** 2007-04-10

**Authors:** S C Pero, G S Shukla, M M Cookson, S Flemer, D N Krag

**Affiliations:** 1 Department of Surgery, Vermont Cancer Comprehensive Center, College of Medicine, University of Vermont, Burlington, VT, USA; 2 Department of Biochemistry, College of Medicine, University of Vermont Protein Core Facility, University of Vermont, Burlington, VT, USA

**Keywords:** Grb7, targeted therapy, non-phosphorylated peptide, breast cancer, trastuzumab, Doxorubicin

## Abstract

Grb7 has potential importance in the progression of cancer. We have previously identified a novel peptide that binds to the SH2 domain of Grb7 and inhibits its association with several different receptor tyrosine kinases. We have synthesised the Grb7 peptide, G7-18NATE, with two different cell penetrating peptides, Penetratin and Tat. In this study, we have shown that both Penetratin- and Tat-conjugated G7-18NATE peptides are able to inhibit the proliferation of SK-BR-3, ZR-75-30, MDA-MB-361 and MDA-MB-231 breast cancer cells. There was no significant effects on breast cancer MCF-7cells, non-malignant MCF 10A or 3T3 cells. In addition, there was no significant inhibition of proliferation by Penetratin or Tat alone or by their conjugates with arbitrary peptide sequence in any of the cell lines tested. We determined the EC_50_ of G7-18NATE-P peptide for SK-BR-3 cell proliferation to be 7.663 × 10^−6^ M. Co-treatment of G7-18NATE-P peptide plus Doxorubicin in SK-BR-3 breast cancer cells resulted in an additional inhibition of proliferation, resulting in 56 and 84% decreases in the Doxorubicin EC_50_ value in the presence of 5 × 10^−6^ and 1.0 × 10^−5^ M G7-18NATE-P peptide, respectively. Importantly, the co-treatment with Doxorubicin and the delivery peptide did not change the Doxorubicin EC_50_. Since Grb7 associates with ErbB2, we assessed whether the peptide inhibitor would have a combined effect with a molecule that targets ErbB2, Herceptin. Co-treatment of Herceptin plus 1.0 × 10^−5^ M G7-18NATE-P peptide in SK-BR-3 cells resulted in a 46% decrease in the Herceptin EC_50_ value and no decrease following the co-treatment with Herceptin and penetratin alone. This Grb7 peptide has potential to be developed as a therapeutic agent alone, in combination with traditional chemotherapy, or in combination with other targeting molecules.

Most chemotherapeutic drugs that are being used presently have little selectivity for cancer cells and target all cells that are rapidly dividing. This leads to increased toxicity against normal cells that have enhanced proliferation, such as bone marrow, gastrointenstinal tract and hair follicles. Since side effects occur as a result of these toxicities to normal tissue, anti-cancer drugs are often given at suboptimal doses, resulting in common failure of the therapy. There are newer drugs being developed against specific molecules that play a key role in the proliferative, migratory and invasive properties of cancer cells.

Many groups have developed growth-inhibitor monoclonal antibodies that target the receptor tyrosine kinase (RTK) ErbB2 (Drebin *et al*, 1985; Hudziak *et al*, 1989; Harwerth *et al*, 1993; Lewis *et al*, 1996). In September 1998, a major clinical milestone in the field of targeted therapeutics was reached when the humanised 4D5 antibody, known as trastuzumab (Herceptin), was approved by the Food and Drug Administration for the treatment of breast cancer (Baselga *et al*, 1996; Slamon and Pegram, 2001). Herceptin binds to the extracellular domain of ErbB2 and induces regression of ErbB2-overexpressing breast cancers (Baselga *et al*, 1996). ErbB2 is an attractive target since it is overexpressed on cancer cells of a number of different histological types and associated with the malignant phenotype. Blocking the function of ErbB2 leads to inhibition of proliferation of cancer cells as demonstrated in preclinical (Hudziak *et al*, 1989; Fendly *et al*, 1990) and clinical studies (Baselga *et al*, 1996; Slamon and Pegram, 2001).

There are a number of clinical trials underway to evaluate the combination treatment of Herceptin with several different chemotherapeutic agents. Herceptin in combination with cisplatin, paclitaxel, docetaxel, gemcitabine and vinorelbine have shown promising clinical results (Yeon and Pegram, 2005; Nahta and Esteva, 2006). The theory is that combining Herceptin with other biological targeting agents may increase the extent of the patient response. Some of the newer novel combinations include Herceptin plus anti-EGFR tyrosine kinase inhibitor gefitinib (Iressa), lapatinib (Tarceva), a combined EGFR/ErbB2 inhibitor lapatinib (GW572016), cyclin-dependent kinase inhibitor flavopiridol, VEGF inhibitor becacizumab (Avastin) and Interleukin inhibitors (Yeon and Pegram, 2005; Nahta and Esteva, 2006).

Grb7 is an adapter-type signalling protein, which is recruited via its SH2 domain to a variety of RTKs, including ErbB2. Grb7 is an especially promising cancer target as the encoding gene maps closely to the ErbB2 gene on human chromosome 17q12 and is found co-amplified and overexpressed in a subset of breast, esophageal and gastric cancers (Stein *et al*, 1994; Kishi *et al*, 1997; Tanaka *et al*, 1997; Fiddes *et al*, 1998; Kauraniemi *et al*, 2001; Varis *et al*, 2002; Andrechek *et al*, 2003; Bieche *et al*, 2003). Analysis of chromosome 17q12 in a variety of different breast cancer cell lines Grb7 was found to have a high DNA copy number with a high level of ErbB2 co-expression (Kauraniemi *et al*, 2001). Similar findings were observed in ErbB2-induced mammary tumours from transgenic mice (Andrechek *et al*, 2003). In addition, Grb7 expression had the strongest association with ErbB2 expression in 54 primary breast tumours analysed by real-time quantitative RT-PCR (Bieche *et al*, 2003). Co-expression of Grb7 with ErbB2 was detected in 31% of esophageal carcinomas and was significantly correlated with extramucosal tumour invasion (Tanaka *et al*, 1997). Moreover, Grb7 is co-expressed with ErbB3 and ErbB4, which are known to heterodimerise with ErbB2, in a subgroup of human breast cancer cell lines. These studies, both *in vitro* and *in vivo*, showing Grb7 being over-expressed or co-overexpressed with ErbB2 emphasises the potential importance of Grb7 in cancer progression.

In our previous studies, we discovered a novel non-phosphorylated peptide that binds to the SH2 domain of Grb7. This peptide was a disulfide-bridged cyclic peptide generated by filamentous phage display (Pero *et al*, 2002). This peptide contained 10 amino acids flanked by two terminal cysteines to create a cyclic peptide through disulfide linkage. The free peptide was synthesised as a redox stable thioether-bridged analog, referred to as G7-18NATE (Pero *et al*, 2002). G7-18NATE was found to specifically bind to the SH2 domain of Grb7 and inhibit the association of Grb7 protein with various RTKs (Pero *et al*, 2002; Tanaka *et al*, 2006). Since Grb7 is an intracellular target, we made G7-18NATE peptide cell permeable by adding the penetratin peptide sequence, referred to as G7-18NATE-P. Penetratin is a peptide derived from the *Drosophila* transcription factor Antennapedia that mediates rapid cellular delivery of proteins and peptides (Deshayes *et al*, 2005). We have shown previously that the cell permeable G7-18NATE-P peptide is able to successfully translocate across the cell membrane and significantly inhibited cell migration and peritoneal metastasis in a pancreatic cancer mouse model (Tanaka *et al*, 2006). In the present study we also tested G7-18NATE conjugated to the Tat cell penetrating peptide. Tat is derived from the transcription factor protein of HIV-1 and known to deliver proteins and peptides across the cell membrane (Deshayes *et al*, 2005).

Since ErbB2 has a role in proliferation of breast cancer cells and Grb7 associates with ErbB2, we are interested in looking at the effects of the Grb7 targeting peptide G7-18NATE-P on proliferation on a variety of different breast cancer cell lines. In addition, we have studied the effects of treating cancer cells with G7-18NATE-P peptide in combination with the chemotherapeutic agent Doxorubicin and Herceptin.

## METHODS

### Peptide synthesis

Peptides were synthesised by the University of Vermont Protein Core Facility. Thioether-cyclised peptides were synthesised according to methodologies described previously (Pero *et al*, 2002). Briefly, the peptides were synthesised on a PAL amide resin using standard solid phase Fmoc chemistry-based protocols. The resin-bound side chain protected peptides were N-terminally chloroacetylated with *in situ*-generated chloroacetic anhydride. The peptides were removed from the resin and fully deprotected with 96 : 2 : 2 trifluoroacetic acid/triisopropylsilane/water. The crude peptides were cyclised by intramolecular nucleophilic displacement of the N-terminal chloro group by the C-terminal cysteine side chain thiol functionality in an aqueous solution (0.1 mmol of peptide in 120 ml of water) maintained at pH 8–8.5 with triethylamine at room temperature for 6 h. The peptides were purified by reverse phase high-pressure liquid chromatography in water/acetonitrile (0.05% trifluoroacetic acid) gradient. The purity of the peptides was confirmed on a Shimadzu analytical high-pressure liquid chromatography system (Shimadzu Corporation; Kyoto, Japan). The identity of the peptides was determined using an ABI Voyager DE-Pro Matrix Assisted Laser-desorption Ionisation instrument (Applied Biosystems; Foster City, CA, USA) under positive ionisation and in reflectron mode. All samples were run using a matrix of 10 mg ml^−1^ alpha-cyano-4-hydroxycinnamic acid vacuum dried from a solution of 1 : 1 acetonitrile buffered to 0.1% trifluoroacetic acid. The peptides amino acid compositions are listed in Table 1. In this study, we synthesised G7-18NATE peptide with two different cell delivery peptides, Penetratin (referred to as G7-18NATE-P) and Tat (referred to as G7-18NATE-T) (Table 1). Additional control peptides were also synthesised; these include Penetratin and Tat alone, as well as arbitrary peptides of the same length with a thioether linkage conjugated to Penetratin (referred to as NegCtrl-P) and Tat (referred to as NegCtrl-T), to mimic the same structure as the G7-18NATE-conjugated peptides.

### Cell culture

Human breast cancer cell lines SK-BR-3, MDA-MB-231, MDA-MB-361, ZR-75-30, MCF-7, non-malignant human mammary epithelial MCF 10A and mouse fibroblast 3T3 cell lines were all obtained from American Type Culture Collection (ATCC; Manassas, VA, USA). All cells were grown according to ATCC instructions except MDA-MB-231 and MDA-MB-361 were grown in Dulbecco's Modified Eagles Medium (ATCC)+10% fetal bovine serum (ATCC) and MCF-7 cells were grown in Dulbecco's Modified Eagles Medium without phenol red (Mediatech, Herndon, VA, USA). All cell lines were grown at 37°C at 5% CO_2_.

### Cell proliferation assays

Proliferation was quantified using the BrdU chemiluminescent cell proliferation ELISA (Roche Applied Science; Indianapolis, IN, USA) following manufacturer's instructions. The 96-well white tissue culture-treated plates (Corning; Acton, MA, USA) were seeded with 1 × 10^4^ cells per well and allowed to adhere for 24 h. Cells were washed three times in Hank's Balanced Salt Solution and shifted to serum-free medium overnight. The next day, cells were treated with different peptides, trastuzumab (Herceptin, Genentech, South San Francisco, CA, USA), and/or Doxorubicin (Sigma; St Louis, MO) and incubated at 37°C, 5% CO_2_ for 8 h. After the 8 h incubation, the BrdU labelling reagent and a second dose of peptide were added to the cells and incubated for an additional 16 h at 37°C, 5% CO_2_. Chemiluminescent signals (rlu/s) were detected on the GloRunner luminometer (Turner Biosystems, Sunnyvale, CA, USA). Percentage of inhibition was calculated as follows: 100−(treated rlu/s÷untreated rlu/s) × 100. Each proliferation experiment was carried out at least twice with triplicate or quadruplicate treatments. The results are described as mean±s.d. of multiple experiments. EC_50_ values were determined with nonlinear regression analysis using the Prism software version 4.03 for Windows (GraphPad Software, San Diego, CA, USA).

## RESULTS

### Cell permeable G7-18NATE peptides inhibit proliferation of breast cancer cells

**Figure 1:**
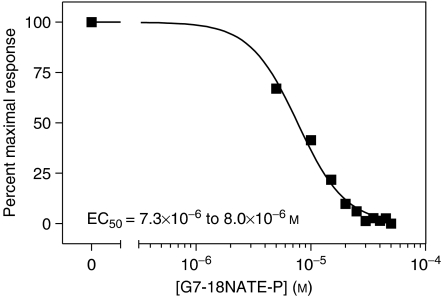
SK-BR-3 cells treated with varying concentrations of G7-18NATE-P to determine EC_50_. Proliferation assays performed using a chemiluminescent BrdU assay. SK-BR-3 breast cancer cells treated with 0 M to 5 × 10^−5^ M, at 5 × 10^−6^ M increments. EC_50_ values were determined with nonlinear regression analysis.

Using a BrdU-based cell proliferation ELISA, the effect of G7-18NATE-P peptide has been evaluated on the proliferation of the SK-BR-3 breast cancer cells. We found significant inhibition of proliferation on SK-BR-3 breast cancer cells treated with the Grb7 targeting peptide, G7-18NATE-P. Figure 1 demonstrates this inhibitory response of G7-18NATE-P with an EC_50_ at 7.3 × 10^−6^– 8.0 × 10^−6^ M (*R*^2^=0.9903).

**Figure 2:**
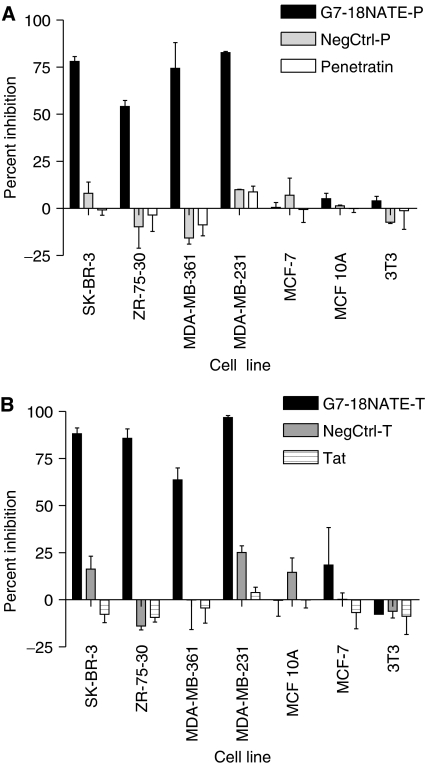
Effects of G7-18NATE-P and G7-18NATE-T peptides on the growth of breast cancer cells and non-malignant cells. Proliferation assays performed using a chemiluminescent BrdU assay. Breast cancer cells tested include SK-BR-3, ZR-75-30, MDA-MB-361, MDA-MB-231 and MCF-7. The non-malignant human breast cells MCF10 A and mouse fibroblast 3T3 cells were also tested. (**A**) Cells treated with 1.0 × 10^−5^ M G7-18NATE-P, NegCtrl-P or Penetratin alone. (**B**) Cells treated with 5 × 10^−5^ M G7-18NATE-T, NegCtrl-T or Tat alone. Sequence of peptides listed in Table 1. Each proliferation experiment was carried out at least twice with triplicate or quadruplicate treatments. Values represent the mean±s.d. of multiple experiments, expressed as a percentage of the growth relative to untreated controls.

In addition to the ErbB2 and Grb7 overexpressing SK-BR-3 cells, we were interested in evaluating the effects of G7-18NATE-P on a variety of different cells; including two additional overexpressing cancer cell lines, ZR-75-30 and MDA-MB-361, two non-overexpressing cell lines, MCF-7 and MDA-MB-231, and two non-malignant cell lines, breast epithelial MCF 10A cells and fibroblast 3T3 cells. Figure 2A demonstrates that 1.0 × 10^−5^ M G7-18NATE-P inhibits 78% of SK-BR-3, 75% of MDA-MB-361 and 83% of MDA-MB-231 cell proliferation. There was slightly less but still significant inhibition for ZR-75-30 cells, which was measured at 54% inhibition of cell proliferation as compared to untreated cells. The MDA-MB-231 is the only breast cancer cell line tested that was found to be sensitive to G7-18NATE-P without overexpression of Grb7. There is no significant inhibition found in the non-overexpressing breast cancer cells line MCF-7 or non-malignant cell lines MCF 10A and 3T3. Moreover, there was no significant inhibition of proliferation by penetratin alone or by NegCtrl-P peptide in any of the cell lines tested (Figure 2A).

To demonstrate that the anti-proliferation effect was a result of the G7-18NATE peptide and not associated with delivery peptide, we tested G7-18NATE conjugated to Tat. Although the effective concentration of the Tat-conjugated G7-18NATE peptide was higher (5.0 × 10^−5^ M), we found similar inhibition of proliferation in the SK-BR-3, ZR-75-30, MDA-MB-361 and MDA-MB-231 cells with no effect in MCF-7, non-malignant MCF 10A or 3T3 cells (Figure 2B). We found G7-18NATE-T peptide inhibited 88% SK-BR-3, 86% ZR-75-30, 64% MDA-MB-361 and 98% MDA-MB-231 cell proliferation. There was no significant inhibition by Tat peptide alone or Tat conjugated to the arbitrary negative control peptide. This series of experiments demonstrates that the G7-18NATE-conjugated peptides are able to significantly inhibit the proliferation of several different breast cancer cells.

### Cell permeable G7-18NATE peptides have a cooperative effect with Doxorubicin and Herceptin

**Figure 3:**
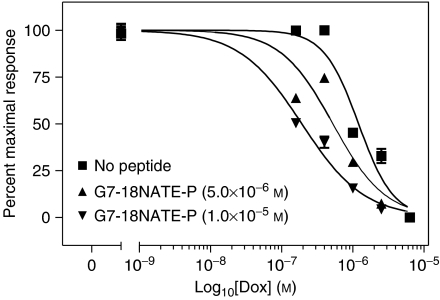
Effects of combined treatment with G7-18NATE-P and Doxorubicin on SK-BR-3 breast cancer cells. Proliferation assays performed using a chemiluminescent BrdU assay. SK-BR-3 breast cancer cells treated with 5.0 × 10^−6^ and 1.0 × 10^−5^ M G7-18NATE-P peptide with varying concentrations of Doxorubicin (1.6 × 10^−7^– 6.3 × 10^−6^ M). Values represent the mean (*n*=3)±s.d. In certain cases, the error bars are smaller than the symbols, therefore they are not visible. EC_50_ values were determined with nonlinear regression analysis.

SK-BR-3 breast cancer cells were treated with Doxorubicin alone or along with G7-18NATE-P peptide. Using non-linear regression analysis, the EC_50_ value was determined for Doxorubicin-treated SK-BR-3 cells to be 1.2 × 10^−6^ M. Co-treatment of Doxorubicin plus G7-18NATE-P peptide resulted in an additional inhibition of proliferation (Figure 3). There was a 56 and 84% lowering of the Doxorubicin EC_50_ value with 5 × 10^−6^ and 1 × 10^−5^ M G7-18NATE-P peptide, respectively. Co-treatment with Doxorubicin and the delivery peptide did not change the Doxorubicin EC_50_ (Table 2). We found similar results with cells treated with Doxorubicin and the Tat-conjugated G7-18NATE peptide (data not shown). This shows that the treatment with G7-18NATE-P increased Doxorubicin-mediated inhibition of cell growth.

**Figure 4:**
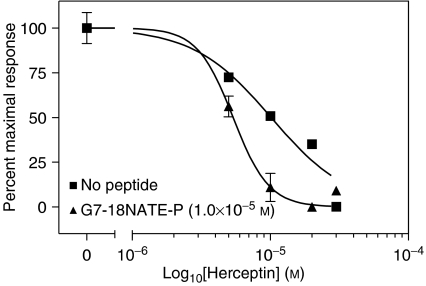
Effects of combined treatment with G7-18NATE-P and Herceptin on SK-BR-3 breast cancer cells. Proliferation assays performed using a chemiluminescent BrdU assay. SK-BR-3 breast cancer cells treated with 1.0 × 10^−5^ M G7-18NATE-P peptide with varying concentrations of Herceptin (1 × 10^−6^–3 × 10^−5^ M). Values represent the mean (*n*=4)±s.d. In certain cases the error bars are smaller than the symbols, therefore they are not visible. EC_50_ values were determined with nonlinear regression analysis.

To assess whether the peptide inhibitor would have a combined effect with a molecule that targets ErbB2, we tested G7-18NATE-P with Herceptin. SK-BR-3 breast cancer cells were treated with Herceptin alone or along with G7-18NATE-P peptide followed by BrdU analysis to measure cell proliferation. Using non-linear regression analysis, the EC_50_ value was determined for Herceptin to be 9.6 × 10^−6^ M. Co-treatment of Herceptin plus 1.0 × 10^−5^ M G7-18NATE-P peptide resulted in a 46% decrease in the Herceptin EC_50_ value (Figure 4). Similar results were obtained with cells treated with Herceptin and the Tat-conjugated G7-18NATE peptide (data not shown). We found no decrease of Herceptin EC_50_ following co-treatment with Herceptin and penetratin alone (Table 3). This series of experiments demonstrates that the Grb7 targeting peptide cooperatively inhibits the proliferation of SK-BR-3 breast cancer cells with Doxorubicin and Herceptin.

## DISCUSSION

Breast cancer is the second leading cause of cancer death in the United States, affecting approximately one in eight women over the course of their lifetime (Ries *et al*, 2005). Tumour size, grade, stage, estrogen receptor expression and lymph node involvement have been the markers used to select the course of therapy. With the recent FDA approval of Herceptin, ErbB2 expression is a key marker being identified in patients. ErbB2 positive patients being treated with Herceptin along with chemotherapy have shown prolonged survival rates. Despite these advances, the clinical benefit of Herceptin is compromised by the fact that not all ErbB2-overexpressing cancers respond clinically to the treatment and some cancers develop resistance to this molecularly targeted drug after initial response. It will be important to identify additional targets for molecularly directed therapies to treat a greater number of patients.

Grb7 is an SH2 containing adapter protein, which is known to associate with several tumour-related molecules (Pero *et al*, 2003). The importance of Grb7 in tumour progression has been suggested by several studies (Tanaka *et al*, 1997, 1998, 2000; Han and Guan, 1999). Downregulating Grb7 either by anti-sense or siRNA technology has shown to inhibit the invasive (Tanaka *et al*, 1998) or proliferative (Kao and Pollack, 2006) properties of cancer cells. Another promising feature of Grb7 as a tumour target is its limited tissue distribution, which is unlike that of many other SH2 domain-containing proteins which are ubiquitously expressed (Margolis, 1994). Having limited tissue distribution is advantageous for targeting therapeutics to cancer cells.

In this study, we have shown that cell permeable Grb7 binding peptides inhibit the proliferation of several different breast cancer cells. We found that G7-18NATE conjugated to Penetratin and Tat has the same profile of inhibition on breast cancer cell proliferation. However, the effective concentration for the Tat-conjugated peptide was higher than the conjugated peptide, which may be due to efficiency of translocation resulting from differences in the mechanism of action of the two penetrating peptides. Inhibition was observed in the SK-BR-3, ZR-75-30, MDA-MB-361 and MDA-MB-231 breast cancer cells. The observation of Grb7 peptide-induced inhibition of MDA-MB-231 cells, which do not overexpress Grb7, demonstrates the peptide does not essentially require Grb7 overexpression and may utilise some other mechanism of inhibition in certain cell types. This type of phenomenon has also been observed with Herceptin. Menendez *et al* (2006) found Herceptin to inhibit certain breast cancer cells that do not overexpress ErbB2 but do overexpress heregulin. Although MDA-MB-231 does not overexpress ErbB2 or Grb7, it does overexpress heregulin and promotes tumourigenicity and metastasis of breast cancer cells (Aguilar and Slamon, 2001). Interestingly, the Grb7 peptide inhibits MDA-MB-231 cells and not MCF-7, which does not overexpress heregulin, suggests there may be a correlation between Grb7 peptide-induced response and heregulin overexpression. Additional experiments will be required to understand the mechanism of action for our Grb7 peptide.

Although we see dramatic inhibition of the proliferation on breast cancer cells following G7-18NATE peptide treatment, we have not been able to detect changes in phosphorylation of ERK or AKT, two known molecules that have a role in cell survival downstream of ErbB2. We have shown previously that the G7-18NATE peptide inhibits the association of Grb7 with the ErbB family of tyrosine kinases (Pero *et al*, 2002) and focal adhesion kinase (Tanaka *et al*, 2006). The signal transduction pathway for Grb7 has not been identified clearly. Therefore, probing into multiple signalling pathways will be necessary to determine the pathway that G7-18NATE peptide is invading.

In this study, we have also shown that the Grb7 peptide inhibitor enhanced the inhibitory effect on SK-BR-3 proliferation when treated with Doxorubicin. The peptide was able to significantly reduce the EC_50_ of Doxorubicin. Most chemotherapeutic drugs are given at suboptimal doses to minimise undesired side effects. Combining chemotherapy with molecules, such as our Grb7 peptide, may have great potential at effectively killing the cancer cells and minimising toxicity. In our recent study we have shown that the Grb7 peptide inhibitor had low toxicity in mice (Tanaka *et al*, 2006). In addition, this peptide is non-phosphorylated, which may have an advantage of stability of other SH2 targeting peptides that possess a highly charged phosphate group. When phosphate groups are attached to a macromolecule, the ability of the compound to enter cells is reduced, and the phosphorylated compound is instable *in vivo* because of the presence of endogenous phosphatases.

Since Grb7 associates with many oncogenic protein tyrosine kinases, such as EGFR (Margolis *et al*, 1992), ErbB2 (Stein *et al*, 1994), Erbb3 (Fiddes *et al*, 1998) and ErbB4 (Fiddes *et al*, 1998), combination therapy with protein tyrosine kinase targeting agents and the Grb7 peptide inhibitor may be a novel therapeutic intervention. Here we show that the Grb7 peptide in combination with Herceptin treatment enhances the inhibitory effect on SK-BR-3 proliferation. It is also worth noting that the Grb7 peptide is able to effectively inhibit the ErbB2 and Grb7 overexpressing MDA-MB-361 cells, which has been described previously as Herceptin resistant (Yakes *et al*, 2002). In conclusion, this Grb7 inhibitory peptide has potential to be developed as a therapeutic agent alone, in combination with traditional chemotherapy, or in combination with other targeting molecules for the treatment of cancer.

## Figures and Tables

**Table 1:** Sequence and names of G7-18NATE peptides and negative control peptides

G7-18NATE-Penetratin (G7-18NATE-P)	WFEGYDNTFPC*RQIKIWFQNRRMKWKK
G7-18NATE-Tat (G7-18NATE-T)	WFEGYDNTFPC*YGRKKRRQRRR
Negative control penetratin (NegCtrl-P)	RQAVSIAQASC*RQIKIWFQNRRMKWKK
Negative control Tat (NegCtrl-T)	RQAVSIAQASC*YGRKKRRQRRR
Penetratin	RQIKIWFQNRRMKWKK
Tat	YGRKKRRQRRR

^*^Cysteine that creates the thioether linkage with the N-terminal residue.

**Table 2:** EC_50_ values of Doxorubicin-treated SK-BR-3 cells with and without peptides

	**Doxorubicin**	**Doxorubicin+5.0 × 10^−6^ M G7-18NATE-P**	**Doxorubicin+1 × 10^−5^ M G7-18NATE-P**	**Doxorubicin+5.0 × 10^−6^ M Penetratin**	**Doxorubicin+1 × 10^−5^ M Penetratin**
Best fit	1.2 × 10^−6^	5.2 × 10^−7^	1.9 × 10^−7^	1.0 × 10^−6^	1.2 × 10^−6^
95% confidence interval	9.7 × 10^−7^–1.5 × 10^−6^	3.9 × 10^−7^– 7.1 × 10^−7^	1.5 × 10^−7^– 2.4 × 10^−7^	9.6 × 10^−7^–1.1 × 10^−6^	1.0 × 10^−6^–1.4 × 10^−6^

**Table 3:** EC_50_ values of Herceptin-treated SK-BR-3 cells with and without peptides

	**Herceptin**	**Herceptin+1 × 10^−5^ M G178NATE-P**	**Herceptin+1 × 10^−5^ M Pen**
Best fit	9.6 × 10^−6^	5.2 × 10^−6^	1.3 × 10^−5^
95% confidence interval	7.8 × 10^−6^–1.2 × 10^−5^	4.6 × 10^−6^– 6.0 × 10^−6^	1.0 × 10^−5^–1.6 × 10^−5^

## References

[bib1] Aguilar Z, Slamon DJ (2001) The transmembrane heregulin precursor is functionally active. J Biol Chem 276: 44099–44107 11495899 10.1074/jbc.M103442200

[bib2] Andrechek ER, Laing MA, Girgis-Gabardo AA, Siegel PM, Cardiff RD, Muller WJ (2003) Gene expression profiling of neu-induced mammary tumors from transgenic mice reveals genetic and morphological similarities to ErbB2-expressing human breast cancers. Cancer Res 63: 4920–4926 12941816

[bib3] Baselga J, Tripathy D, Mendelsohn J, Baughman S, Benz CC, Dantis L, Sklarin NT, Seidman AD, Hudis CA, Moore J, Rosen PP, Twaddell T, Henderson IC, Norton L (1996) Phase II study of weekly intravenous recombinant humanized anti-p185HER2 monoclonal antibody in patients with HER2/neu-overexpressing metastatic breast cancer. J Clin Oncol 14: 737–744 8622019 10.1200/JCO.1996.14.3.737

[bib4] Bieche I, Onody P, Tozlu S, Driouch K, Vidaud M, Lidereau R (2003) Prognostic value of ERBB family mRNA expression in breast carcinomas. Int J Cancer 106: 758–765 12866037 10.1002/ijc.11273

[bib5] Deshayes S, Morris MC, Divita G, Heitz F (2005) Cell-penetrating peptides: tools for intracellular delivery of therapeutics. Cell Mol Life Sci 62: 1839–1849 15968462 10.1007/s00018-005-5109-0 PMC11139131

[bib6] Drebin JA, Link VC, Stern DF, Weinberg RA, Greene MI (1985) Down-modulation of an oncogene protein product and reversion of the transformed phenotype by monoclonal antibodies. Cell 41: 697–706 2860972 10.1016/s0092-8674(85)80050-7

[bib7] Fendly BM, Winget M, Hudziak RM, Lipari MT, Napier MA, Ullrich A (1990) Characterization of murine monoclonal antibodies reactive to either the human epidermal growth factor receptor or HER2/neu gene product. Cancer Res 50: 1550–1558 1689212

[bib8] Fiddes RJ, Campbell DH, Janes PW, Sivertsen SP, Sasaki H, Wallasch C, Daly RJ (1998) Analysis of Grb7 recruitment by heregulin-activated erbB receptors reveals a novel target selectivity for erbB3. J Biol Chem 273: 7717–7724 9516479 10.1074/jbc.273.13.7717

[bib9] Han DC, Guan JL (1999) Association of focal adhesion kinase with Grb7 and its role in cell migration. J Biol Chem 274: 24425–24430 10446223 10.1074/jbc.274.34.24425

[bib10] Harwerth IM, Wels W, Schlegel J, Muller M, Hynes NE (1993) Monoclonal antibodies directed to the erbB-2 receptor inhibit *in vivo* tumour cell growth. Br J Cancer 68: 1140–1145 7903153 10.1038/bjc.1993.494 PMC1968669

[bib11] Hudziak RM, Lewis GD, Winget M, Fendly BM, Shepard HM, Ullrich A (1989) p185HER2 monoclonal antibody has antiproliferative effects *in vitro* and sensitizes human breast tumor cells to tumor necrosis factor. Mol Cell Biol 9: 1165–1172 2566907 10.1128/mcb.9.3.1165-1172.1989 PMC362707

[bib12] Kao J, Pollack JR (2006) RNA interference-based functional dissection of the 17q12 amplicon in breast cancer reveals contribution of coamplified genes. Genes Chromosomes Cancer 45: 761–769 16708353 10.1002/gcc.20339

[bib13] Kauraniemi P, Barlund M, Monni O, Kallioniemi A (2001) New amplified and highly expressed genes discovered in the ERBB2 amplicon in breast cancer by cDNA microarrays. Cancer Res 61: 8235–8240 11719455

[bib14] Kishi T, Sasaki H, Akiyama N, Ishizuka T, Sakamoto H, Aizawa S, Sugimura T, Terada M (1997) Molecular cloning of human GRB-7 co-amplified with CAB1 and c-ERBB-2 in primary gastric cancer. Biochem Biophys Res Commun 232: 5–9 9125150 10.1006/bbrc.1997.6218

[bib15] Lewis GD, Lofgren JA, McMurtrey AE, Nuijens A, Fendly BM, Bauer KD, Sliwkowski MX (1996) Growth regulation of human breast and ovarian tumor cells by heregulin: evidence for the requirement of ErbB2 as a critical component in mediating heregulin responsiveness. Cancer Res 56: 1457–1465 8640840

[bib16] Margolis B (1994) The GRB family of SH2 domain proteins. Prog Biophys Mol Biol 62: 223–244 7892504 10.1016/0079-6107(94)90013-2

[bib17] Margolis B, Silvennoinen O, Comoglio F, Roonprapunt C, Skolnik E, Ullrich A, Schlessinger J (1992) High-efficiency expression/cloning of epidermal growth factor-receptor-binding proteins with Src homology 2 domains. Proc Natl Acad Sci USA 89: 8894–8898 1409582 10.1073/pnas.89.19.8894 PMC50030

[bib18] Menendez JA, Mehmi I, Lupu R (2006) Trastuzumab in combination with heregulin-activated Her-2 (erbB-2) triggers a receptor-enhanced chemosensitivity effect in the absence of Her-2 overexpression. J Clin Oncol 24: 3735–3746 16847284 10.1200/JCO.2005.04.3489

[bib19] Nahta R, Esteva FJ (2006) Herceptin: mechanisms of action and resistance. Cancer Lett 232: 123–138 16458110 10.1016/j.canlet.2005.01.041

[bib20] Pero SC, Daly RJ, Krag DN (2003) Grb7-based molecular therapeutics in cancer. Expert Reviews in Molecular Medicine 5: 1–11 10.1017/S1462399403006227 14585167

[bib21] Pero SC, Oligino L, Daly RJ, Soden AL, Liu C, Roller PP, Li P, Krag DN (2002) Identification of novel non-phosphorylated ligands, which bind selectively to the SH2 domain of Grb7. J Biol Chem 277: 11918–11926 11809769 10.1074/jbc.M111816200

[bib22] Ries LAG, Eisner MP, Kosary CL, Hankey BF, Miller BA, Clegg L, Mariotto A, Feuer EJ, Edwards BK (2005) SEER Cancer Statistics Review, 1975–2002. Bethesda, MD: National Cancer Institute

[bib23] Slamon D, Pegram M (2001) Rationale for trastuzumab (Herceptin) in adjuvant breast cancer trials. Semin Oncol 28: 13–19 10.1016/s0093-7754(01)90188-5 11301370

[bib24] Stein D, Wu J, Fuqua SA, Roonprapunt C, Yajnik V, D'Eustachio P, Moskow JJ, Buchberg AM, Osborne CK, Margolis B (1994) The SH2 domain protein GRB-7 is co-amplified, overexpressed and in a tight complex with HER2 in breast cancer. EMBO J 13: 1331–1340 7907978 10.1002/j.1460-2075.1994.tb06386.x PMC394949

[bib25] Tanaka S, Mori M, Akiyoshi T, Tanaka Y, Mafune K, Wands JR, Sugimachi K (1997) Coexpression of Grb7 with epidermal growth factor receptor or Her2/erbB2 in human advanced esophageal carcinoma. Cancer Res 57: 28–31 8988034

[bib26] Tanaka S, Mori M, Akiyoshi T, Tanaka Y, Mafune K, Wands JR, Sugimachi K (1998) A novel variant of human Grb7 is associated with invasive esophageal carcinoma. J Clin Invest 102: 821–827 9710451 10.1172/JCI2921 PMC508945

[bib27] Tanaka S, Pero SC, Taguchi K, Shimada M, Mori M, Krag DN, Arii S (2006) Specific peptide ligand for Grb7 signal transduction protein and pancreatic cancer metastasis. J Natl Cancer Inst 98: 491–498 16595785 10.1093/jnci/djj105

[bib28] Tanaka S, Sugimachi K, Kawaguchi H, Saeki H, Ohno S, Wands JR (2000) Grb7 signal transduction protein mediates metastatic progression of esophageal carcinoma. J Cell Physiol 183: 411–415 10797316 10.1002/(SICI)1097-4652(200006)183:3<411::AID-JCP14>3.0.CO;2-Z

[bib29] Varis A, Wolf M, Monni O, Vakkari ML, Kokkola A, Moskaluk C, Frierson Jr H, Powell SM, Knuutila S, Kallioniemi A, El-Rifai W (2002) Targets of gene amplification and overexpression at 17q in gastric cancer. Cancer Res 62: 2625–2629 11980659

[bib30] Yakes FM, Chinratanalab W, Ritter CA, King W, Seelig S, Arteaga CL (2002) Herceptin-induced inhibition of phosphatidylinositol-3 kinase and Akt is required for antibody-mediated effects on p27, cyclin D1, and antitumor action. Cancer Res 62: 4132–4141 12124352

[bib31] Yeon CH, Pegram MD (2005) Anti-erbB-2 antibody trastuzumab in the treatment of HER2-amplified breast cancer. Invest New Drugs 23: 391–409 16133791 10.1007/s10637-005-2899-8

